# PRESEE: An MDL/MML Algorithm to Time-Series Stream Segmenting

**DOI:** 10.1155/2013/386180

**Published:** 2013-06-20

**Authors:** Kaikuo Xu, Yexi Jiang, Mingjie Tang, Changan Yuan, Changjie Tang

**Affiliations:** ^1^College of Computer Science & Technology, Chengdu University of Information Technology, Chengdu 610225, China; ^2^School of Computing and Information Sciences, Florida International University, Miami, IN 33199, USA; ^3^Department of Computer Science, Purdue University, West Lafayette, FL 47996, USA; ^4^Guangxi Teachers Education University, Nanning 530001, China; ^5^School of Computer Science, Sichuan University, Chengdu 610065, China

## Abstract

Time-series stream is one of the most common data types in data mining field. It is prevalent in fields such as stock market, ecology, and medical care. Segmentation is a key step to accelerate the processing speed of time-series stream mining. Previous algorithms for segmenting mainly focused on the issue of ameliorating precision instead of paying much attention to the efficiency. Moreover, the performance of these algorithms depends heavily on parameters, which are hard for the users to set. In this paper, we propose *PRESEE* (*parameter-free, real-time, and scalable time-series stream segmenting algorithm*), which greatly improves the efficiency of time-series stream segmenting. PRESEE is based on both MDL (minimum description length) and MML (minimum message length) methods, which could segment the data automatically. To evaluate the performance of PRESEE, we conduct several experiments on time-series streams of different types and compare it with the state-of-art algorithm. The empirical results show that PRESEE is very efficient for real-time stream datasets by improving segmenting speed nearly ten times. The novelty of this algorithm is further demonstrated by the application of PRESEE in segmenting real-time stream datasets from ChinaFLUX sensor networks data stream.

## 1. Introduction

Time series stream is everywhere in our daily life. It is widely used in fields such as ecology, medical care, and environment. These applications make time series stream type be possibly the most frequently encountered type for data mining problems [1]. Hence, in recent years, a large number of works focus on time series stream mining.

**Figure 1:**
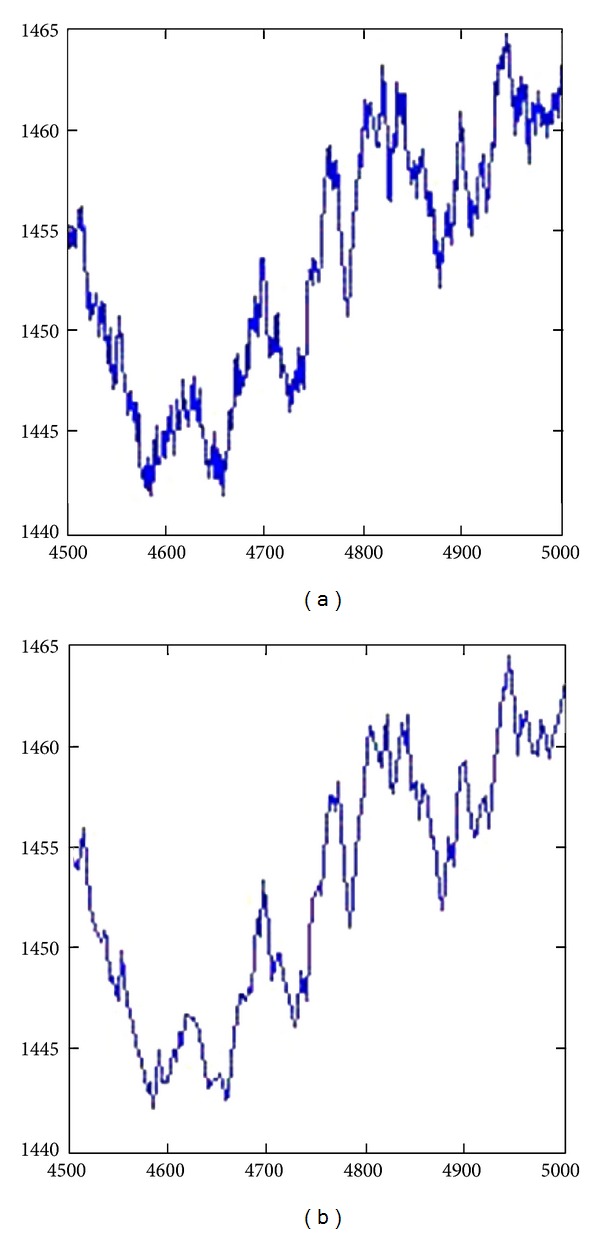
Time series original data (a) and its segmenting result (b).

In order to process massive data efficiently, the method of time series stream segmenting is employed. The primary purpose of time series segmenting is dimensionality reduction. To achieve the goal of accelerating later mining tasks, time-series stream segmenting decomposes the time series stream into smaller number of segments. After segmenting, each segment can be described by a simple model like linear segment and monotonic segment [2]. An example of time-series segmenting can be seen in Figure 1.

There are several time series stream fitting models proposed, including symbolic mappings [3], adaptive multivariate spline [4], hybrid adaptive [5], wavelets [6], Fourier transforms [7], and piecewise linear representation [8, 9]. However, neither of them could handle different types of time series streams or is parameter free.

For real-time series application, the algorithm should be able to handle continuously real-time stream, which means that the stream could only be scanned once. A lot of real applications such as sensor network data [10], stock market trading data [11], or intensive-care unit (ICU) data [12] are in this form since the data are generated very fast and the processing time is limited. So, for time series stream segmentation, the issues of scalability, numerical stability, and efficiency cannot be avoided.

In this paper, we propose PRESEE to segment time series stream based on MDL/MML method [13, 14]. MDL/MML is an information expressing method in the field of information theory. By capturing the characteristics of information distribution in data, it can reduce the size of data while retaining most of the critical information. PRESEE the following characteristics has

1. High scalability. It can process time series stream in linear time. PRESEE adopts slide window to process data with the size of gigabytes or even larger scale.
2. Parameter free. Parameter settings are not essential in PRESEE for an entry-level user. This may avoid the trouble of misleading the algorithm by setting any improper parameters. Of course, if the end users are the domain experts and have confidence to set proper parameters, they can set some optional parameters to accelerate the segmenting speed.
3. Adaptive. PRESEE can segment the time series data according to the characteristic of data. Since the segmenting strategy is based on MDL/MML, it can segment time series automatically. Violating place requires more characteristic points while elsewhere requires less.
4. Pipeline. PRESEE can output the earlier data while processing the newly arrived data. Thus, the later time series stream mining algorithm and PRESEE can run simultaneously.

The rest of this paper is organized as follows. The related work is described in Section 2. Some necessary concepts are introduced in Section 3. A time series stream segmenting algorithm named PRESEE is presented in Section 4. The result of experiments is evaluated in Section 5. Finally, the paper is concluded and future work is discussed in Section 6.

## 2. Related Work

### 2.1. Time Series Stream Mining

Time series stream mining is possibly the most frequent mining task in recent data mining community. In particularly, in the last several years, a large number of papers are related to this area [15–17]. Time series stream mining derives from traditional time-series mining [1–4, 11, 18]. As a further requirement of deep understanding of the time series, it turns into high-dimensional data mining problem. 

#### 2.1.1. Segmenting

Segmenting is one of the major tasks in time-series stream mining. In order to process time-series data efficiently and effectively, segmenting is a key step for other time-series mining tasks. A lot of algorithms focus on finding good global segmenting of the time-series data.

There are mainly three characteristics of these algorithms. Firstly, these methods are mainly based on dynamic programming [19, 20], top-down [21], and bottom-up [22] strategies. Secondly, they require domain expert knowledge to set the parameters, either the parameter to measure the error [2, 22] or parameter *k* (*k* ≪ *n*) to control the number of segments [19, 21]. Thirdly, these algorithms can at most handle millions of data, and they can hardly to handle stream data (gigabytes at least) due to the limitation of the algorithms.

Segmenting with slide window can handle large-scale data. This method is attractive because it can be easily implemented as an online algorithm. Some existing slide-window-based algorithms work well, but their performances are parameter dependent. Since different time-series data types such as electrocardiogram (ECG), water level, and stock market own quite different characteristics, it is hard to find a general set of parameters for all these data types. 

#### 2.1.2. MDL/MML

The theory of minimum message length (MML) and minimum description length (MDL) first appears in the computation complexity community [23, 24] then in the categorization community [25]. Its application in data mining community is the work of climate data segmentation [26], trajectory clustering [27], and social network mining [28]. So far, to the best of our knowledge, our work segmenting time-series stream with MDL/MML is the work with the most features.

## 3. Preliminary

This section reviews the concepts for time-series data mining.  Section 3.1 introduces terminology about the time series.  Section 3.2 presents the distance function used in this paper.  Section 3.3 is the problem statement.

### 3.1. Terminology

We first begin with the definition of the time-series data type.

**Definition 1**

Time-series: let *R*_*d*_ denote a set of the observed values for given variables in the research domain. Let *s*_*i*_∈*R*_*d*_ be an element observed at time *i*. Time-series *S* = 〈*s*_1_ *s*_2_ *s*_3_ ⋯ *s*_*n*_〉 is an ordered sequence of *n* such elements. From the stream view, the length of *S* is infinite.

Slide window may be a general and effective way to handle massive data that cannot be processed in whole. Thus we employ slide window idea to do the segmenting task.

**Definition 2**

Slide window: let *B* be a user-defined buffer to hold elements and *w* be the size of elements that *B* can hold. The slide window *W* = 〈*w*_1_ *w*_2_ ⋯ *w*_*w*_〉 is the buffer to hold a continuous subsequence of *S* at any time. All the data in slide window can be processed by the algorithm in one time.

### 3.2. Distance Function for Time Series Segments

For the ease of segmenting, some data transformation work should be done. Almost all kinds of time series data can be discretized and transformed in the form of lines. For example, the original time-series data *S* = 〈*s*_1_ *s*_2_ ⋯ *s*_*n*_〉 can be discretized into *n* − 1 lines: *s*_1_ *s*_2_,  *s*_2_ *s*_3_, …,  *s*_*n*−1_ *s*_*n*_. The goal of segmenting is to generate *m*  (*m* < *n*) lines that can represent most of the characteristics of original lines. There should be a distance function to measure the distance between the original time-series line *L*_*o*_ and the candidate segment *L*_*s*_. In order to better measure the distance between original time-series stream and its segmenting result, firstly, the distance function should be simple so that the stream can be processed very fast. Additionally, the measurement should consider the shape of stream and its segmenting result. Finally, the focus of factor in measurement can vary according to different application. After delving into the character of time-series data, we find that the best way to measure the distance between time-series by considering the conciseness and preciseness is to use *Hausdorff metric*. *Hausdorff metric* has been previously used in the area of pattern recognition and trajectory mining [27, 29]. Previous works proved that it is precise in the scenario of shape similarity measurement. In the scenario of time-series segmenting, we represent the *segmenting distance* by considering the perpendicular and angle space relationship based on *Hausdorff metric*.

*Segmenting distance* is a quantitative criterion to measure the quality of segmenting. Smaller distance represents better segment result for the original stream. The final form of distance between the original line and the segment it belongs to is defined in Definition 3.

**Definition 3**

*Segmenting distance.* Let *L*_*o*_ be the original line, *L*_*s*_ be the candidate segment, *l*_*p*1_ and *l*_*p*2_ be the distances from the start point and end point of *L*_*o*_ to *L*_*s*_, respectively (Formula (1)), and *θ* (*L*_*o*_, *L*_*s*_) be the smaller intersection angle between two lines. Then the following can be considered.

1. The perpendicular distance between two lines is defined as *d*_*p*_(*L*_*o*_, *L*_*s*_) in Formula (2). In Formula (1), (*x*_*ps*_, *y*_*ps*_) and (*x*_*pe*_, *y*_*pe*_) represent the start point and end point of each original time-series line, respectively; (*x*_*s*_, *y*_*s*_) and (*x*_*e*_, *y*_*e*_) represent the coordinates of the start point and end point of a candidate segment time-series line (one possible segment solution in the process of segmenting computation), respectively.
2. The angle distance between two lines is defined as *d*_*a*_(*L*_*o*_, *L*_*s*_) in Formula (3).
3. The segmenting distance between two lines is defined as *d*(*L*_*o*_, *L*_*s*_) in Formula (4): the weighted sum of perpendicular distance and angle distance.

Consider

(1) $$\begin{array}{c} l_{p1(2)}=\frac{\left|k\times x_{ps(pe)}-y_{ps(pe)}+b\right|}{\sqrt{k^{2}+1}},\\ \left(k=\frac{y_{s}-y_{e}}{x_{s}-x_{e}},b=y_{s}-k\times x_{s}\right), \end{array}$$

(2) $$\begin{array}{c} d_{p}\left(L_{o},L_{s}\right)=\frac{l_{p1}^{2}+l_{p2}^{2}}{l_{p1}+l_{p2}}, \end{array}$$

(3) $$\begin{array}{c} d_{a}\left(L_{o},L_{s}\right)=L_{o}\times \sin\left(\theta \left(L_{o},L_{s}\right)\right), \end{array}$$

(4) $$\begin{array}{c} d\left(L_{o},L_{s}\right)=w_{p}\cdot d_{p}+w_{a}\cdot d_{a}. \end{array}$$

**Figure 2:**
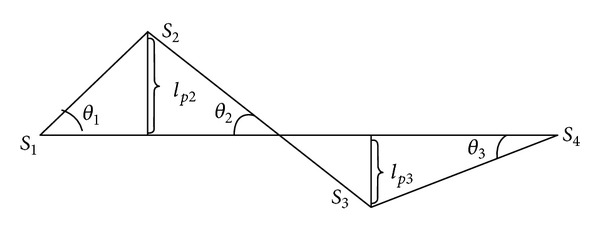
Distance between 3 original lines and one segment that they belong to.

The sum of weight *w*_*p*_ and *w*_*a*_ should be 1, and they can both be set to 1/2 if there is no special requirement.  Figure 2 and Example 4 show an example of how to compute the distance.

**Example 4**

As shown in Figure 2, there are 3 original lines *L*_*o*1_(line *s*_1_ *s*_2_), *L*_*o*2_(line *s*_2_ *s*_3_), and *L*_*o*3_(line *s*_3_ *s*_4_) and one segment *L*_*s*_(line *s*_1_ *s*_4_). Since it can be observed that the start point of *L*_*o*1_ and *L*_*s*_ is the same point, the distance *d*(*L*_*o*1_, *L*_*s*_) = *w*_*p*_ · *l*_*p*2_ + *w*_*a*_ · (*L*_*o*_ · sin*θ*_1_). The distance between *L*_*o*2_ and *L*_*s*_ is *d*(*L*_*o*2_, *L*_*s*_) = *w*_*p*_ · (*l*_*p*2_^2^ + *l*_*p*3_^2^)/(*l*_*p*2_ + *l*_*p*3_) + *w*_*a*_ · (*L*_*o*_ · sin*θ*_2_), and between *L*_*o*3_ and *L*_*s*_ is *d*(*L*_*o*3_, *L*_*s*_) = *w*_*p*_ · *l*_*p*3_ + *w*_*a*_ · (*L*_*o*_ · sin*θ*_3_).

### 3.3. Problem Statement

**Figure 3:**
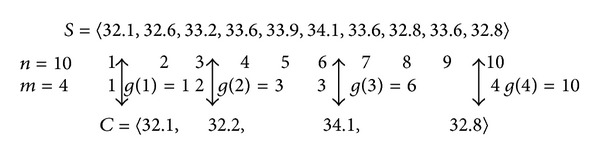
The comparison between original time-series *S* and the character points *C*.

Given a time series *S* with length *n* (i.e., *S* = 〈*s*_1_ *s*_2_ *s*_3_ ⋯ *s*_*n*_〉, *n* can be infinite), our algorithm generates a sequence of character points *C* = 〈*c*_1_, *c*_2_, *c*_3_ … *c*_*m*_〉. For sequence *C*, each pair *c*_*i*_ to *s*_*j*_ has a projection relationship: *g*(*i*) = *j*. This means that each *c*_*i*_ located at *i* in *C* has a counterpart located at *j* in *S*. For each consecutive character point *c*_*x*_, *c*_*y*_ ∈ *C*, there exist several points (*s*_*i*_, …, *s*_*j*_) in *S* such that *g*(*x*) < *i* < *j* < *g*(*y*). Every pair of *c*_*x*_, *c*_*y*_ represents a segment which is an approximation of lines represented by several pairs of consecutive points in the original time series stream *S*. Thus, these *m* character points partition the original stream into *m* − 1 continuous segments. And for each *s*_*x*_ ∈ *S*, if *s*_*x*_ is just the start or end point of one segment, it belongs to two segments; otherwise, it only belongs to one segment.  Figure 3 shows lines representing *S* compared with lines representing *C*.

**Figure 4:**
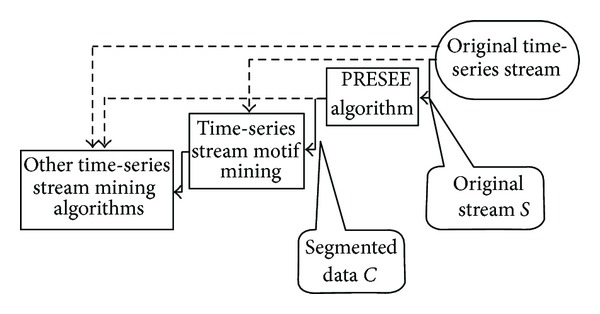
The pipeline for time-series mining. All data are processed as stream. At first the original data are flowed into PRESEE and then flowed into any other time-series stream mining algorithms.

The segmenting algorithm is implemented under a pipeline framework shown in Figure 4. Besides the segmenting algorithm, we had already implemented the time-series stream motif mining algorithm. This framework is designed specifically for handling time-series stream mining. It owns several advantages as follows.

1. Data stream is only scanned once. When data flow out of the slide window, it would never turn back to slide window again.
2. Mining tasks can be processed simultaneously. Earlier data that have been segmented before can be processed by following mining task while the later data is under processing by segmenting task.

## 4. PRESEE Algorithm

This section first introduces how to use segmenting strategy based on MDL/MML in our algorithm PRESEE, and then introduces this algorithm in detail.

### 4.1. Information-Theory-Based Segmenting Strategy

Our algorithm aims at finding the best segments for time-series. As for the problem of segmenting, there are two properties to measure the quality: *preciseness* and *conciseness*. *Preciseness* measures the distance between the lines represented by consecutive points of the character point *c*_*i*_, *c*_*j*_ in set *C* and lines represented by consecutive points of original time series in stream *S* between the corresponding two character points *c*_*i*_, *c*_*j*_. Smaller distance indicates better *preciseness*. *Conciseness* measures how less the character points are used to depict the certain length of data points in original stream. Less character points represents better conciseness.

It is easy to get the conclusion that, when every point in original stream is the character point, *preciseness* gets its maximum. However, such kind of segmenting is meaningless since it in fact just lets the stream go through the slide window and does not do any work to compress the stream. *Conciseness* reaches the maximum when there are only two character points for the stream, the start point and the end point.

The best *preciseness* and best *conciseness* cannot be satisfied at the same time because they are contradictory. Therefore, we need to do some work to find the optimum tradeoff   betwee*n preciseness* and *conciseness*, which generates the best segmenting *L*_opt_.

In order to find the optimum tradeoff, we intend to solve this problem in information perspective by employing the MDL/MML principle in information theory area. We use MDL/MML in our algorithm because it is parameter free. MDL/MML can automatically find a proper estimate of original information. If no proper segmenting solution exists, the data *S* are deemed as random data. In our scenario, we simply keep all the information.

The code of MDL/MML is composed of two parts: *I* = *H* : *D* [30, 31]. *H* specifies the hypothesis about the information (normally selected from a limited set of possible hypotheses), while *D* specifies the code for the information based on the hypothesis. The shortest code *I* in all the *H* and *D* combinations are the optimum solution for the piece of information. In our scenario, it are the optimum for stream segmenting.

The cost of code is represented by its length. In *Shannon's theory*, the length of coding an event *E* in optimum condition is given by −log⁡_2_(*E*). In time-series segmenting scenario, the computation of the formula is as follows:

(5) $$\begin{array}{c} L\left(H\right)=\overset{w-1}{\underset{i=1}{\sum }}\text{lo}{\text{g}}_{2}\left(len\left(s_{c_{i}}s_{c_{i+1}}\right)\right), \end{array}$$

(6) L(S ∣ H)=∑i=1w−1 ∑k=cici+1−1{log2wpdp(scisci+1,sksk+1)+  log2wada(scisci+1,sksk+1)},

(7) $$\begin{array}{c} L_{\text{opt}}=\text{argmin}\left\{L\left(H\right)+L\left(S\;|\;H\right)\right\}. \end{array}$$

In the first two formulas, *w* represents the size of data *S*, *c*_*x*_ ∈ *C* represents the character points. The optimum segmenting is the minimum value of sum of *L*(*H*) and *L*(*S* | *H*). The following is a concrete computation example for Figure 3. Line *s*_1_ *s*_4_ is the optimum segmenting for point *s*_1_ through *s*_4_.

Consider

(8) L(H)=log2(len(s1s4)),L(S ∣ H)={log212dp(s1s2,s1s4)+log212da(s1s2,s1s4)}+{log212da(s2s3,s1s4)+log212dp(s2s3,s1s4)}+{log212da(s3s4,s1s4)+log212dp(s3s4,s1s4)}.

### 4.2. Algorithm Details

Finding global optima requires computing all partitions possibilities of the points, which is prohibitive for real applications. We present a greedy algorithm to find local optima. 

Algorithm 1 shows the details of the segmenting process. At first, only the data flowed into slide window are processed. In lines 5 and 6, the costs of MDL_seg_ and MDL_noseq_ are computed, respectively. MDL_seq_ (*s*_*i*_, *s*_*j*_) denotes the MDL cost by considering *s*_*i*_ and *s*_*j*_ as the character points for points *s*_*x*_ (*i* < *x* < *j*). It equals to *L*(*H*) + *L*(*D* | *H*). MDL_noseq_(*s*_*i*_, *s*_*j*_) denotes the MDL cost by assuming that there are no character points between *s*_*i*_ and *s*_*j*_. It equals to *L*(*H*). In the greedy strategy, the local optimum solution is the longest segment that satisfies the inequality (9).

In the algorithm shown previously, the points in slide window are scanned sequentially only once. The candidate segmenting (segment with *s*_*startIndex*_ and *s*_*curIndex*_ as start point, and end point resp.) grows once per time to test whether it satisfies inequality (9). 

There is a parameter *batchSize* for this algorithm. The default value is 1, and the user can set it as a larger integer. The algorithm will return *batchSize* + 1 character points per time. Thus, the algorithm can run faster.

Consider

(9) $$\begin{array}{c} {\text{MDL}}_{\text{seq}}\left(s_{i},s_{j}\right)<{\;\text{MDL}}_{\text{noseq}}\left(s_{i},s_{j}\right). \end{array}$$

PRESEE algorithm calls Algorithm 1 every time when slide window is full.  Algorithm 2 describes PRESEE algorithm in the form of pseudocode.

In line 2, the slide window is filled at the first time. Then the method *ReadIn*() is called in *while* loop. *ReadIn*() takes the response of filling slide window and checking whether there is new data. It returns *false* when no new data exists. In line 4, Algorithm 1 is called to provide the local optima segmenting result based on the data in slide window. It is possible that no proper segment exists. Thus the size of *tmpSet* is less than 2. In this scenario, we simply put all the data in slide window into *apprSet* and empty the slide window. Otherwise, add *at most* first *batchSize* + 1 points in *tmpSet* into *apprSet*. We use “at most” here because it is possible that all the points in slide window may be generated less than in *batchSize* segments.

From the pseudocode, we can see that the data are input and output simultaneously (line 4 and line 13), which guarantees that the earlier data can be processed by later mining algorithm. Additionally, it is obvious that the stream is only scanned once and processed once. Thus the time complexity of both algorithms is *O*(*n*), where *n* is the length of time-series stream.

## 5. Empirical Comparison of the Segmenting Algorithms

In this section, we demonstrate the effectiveness and efficiency of the proposed algorithm through several sets of experiments on large collections of real and synthetic time-series datasets. For the effectiveness test, the precision of the proposed method is compared with nonstream segmenting algorithm. Then the speed and scalability of the algorithm are tested with a different scale of datasets ranging from 10 M to 10 G.

All the experiments are performed on a laptop computer with 2 GHz Intel Core 2 Duo CPU and 3G main memories. The C++ implementation of the algorithm and the related source code are all available at http://code.google.com/p/otsm/.

### 5.1. Benchmark Algorithm

The performance of the proposed algorithm is compared with well-known benchmark algorithms. A good candidate is the BU (bottom-up) algorithm [22]. It is used as a counterpart in precision algorithm. Since BU cannot handle stream-like datasets and the time complexity is uncertain for large datasets, we use slide-window-based bottom-up (SWBU for short) segmenting algorithm in the part of efficiency and scalability experiment instead.

### 5.2. Dataset. Real Datasets

We consider two sets of real datasets to evaluate our algorithm. The first set includes three classical datasets: IBM stock price dataset from 1.2.1961 to 1.6.2010 [32], “Dodgers,” and “ICU” from UCI Machine Learning Repository [33] used by most of the papers in data mining and machine learning community. The second set of real-time stream data is collected from Chinese Terrestrial Ecosystem Flux Research Network (ChinaFLUX) [34], which is a long-term national network of micrometeorological flux measurement sites that measures the net exchange of carbon dioxide, water vapor, and energy. In this paper, we only choose part of the flux data from one wild filed survey site located in Yucheng, Shandong, China. The data is stored from 2009-03-04 to 2009-11-12 with the number of 38850980.

*Synthetic Datasets.* The synthetic datasets are generated according to Formula (10) with parameters in Table 1. The data is in the format of index, value, where index represents the timestamp of the record. The monotonically increasing record serial number is also available. In our experiments, without loss of generalization, we simply use the latter one.

For synthetic data, the range of values is bounded within [*lb*, *ub*]. The current record value fluctuates (increases, decreases, or remains the same) according to previous record value.

Consider

(10) Δ=sign⁡×(random()mod⁡(lb−previous_value))×sharpness.

The *sign* is randomly selected as either + or −, and *sharpness* is a parameter to control the power of fluctuation. It is easy to observe that when the value would suffer more resistance when it goes far away from the mean ((*ub* − *lb*)/2).

## 6. Results

**Figure 5:**
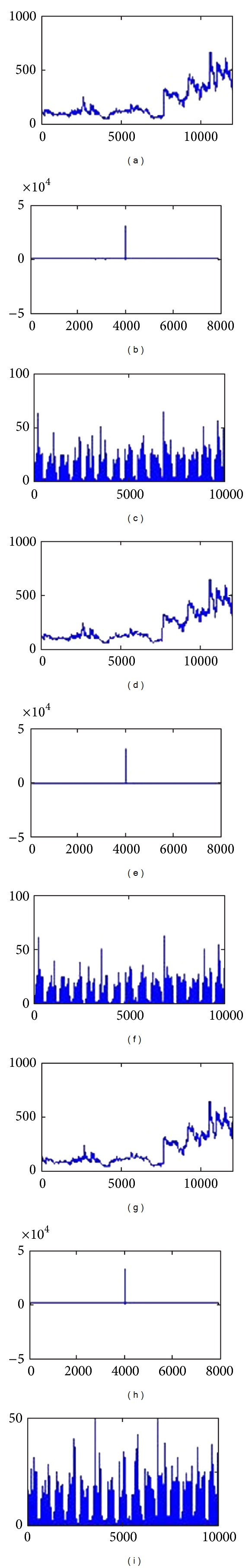
Time-series streams with their segmenting results: (a) IBM stock price, (b) ICU, and (c) Dodger. (d)–(f) Segmenting result with bottom-up. (g)–(i) Segmenting result with PRESEE.

*Visualization of Segmenting Result.* For the ease of observing the segmenting result, Figure 5 presents the visualization of three real datasets (IBM stock, ICU, and Dodger). The first row is the original datasets; the second row is the segmenting result generated by BU; and the last row is generated by PRESEE. It is obvious that the charts in the first row seem to be the most complex because they contain the most detailed information about the time-series. For the other two rows, the charts seem to be more concise because segmenting algorithm removes some of the unimportant information from original time-series. Nevertheless, the main trends in the charts are reserved. This means that both algorithms keep the characteristics of original time-series data.

*Precision. *We compare our algorithm with BU, which can process general types of time-series datasets and can find the global optimum segmenting solution. We evaluate the result of segmenting algorithm via the* error rate *measure in Formula (11). Let *w* be the number of segments and *n* be the number of points in segment *i*. The error rate is computed as follows:

(11) $$\begin{array}{c} \text{error}\_\;\text{rate}=\frac{1}{w}\overset{i<w}{\underset{i=1}{\sum }}\frac{1}{n}\;\overset{}{\underset{o_{j}\in s_{i}}{\sum }}d\left(L_{o_{j}},L_{s_{i}}\right)\text{.} \end{array}$$

*Error rate *for fifteen datasets is reported in Table 2. It is evident that, for generating approximately the same number of segments, the error rate of bottom-up and PRESEE stays in the same level. That means that PRESEE can generate segments based on partial data (data only in slide window) no worse than segments generated in global perspective.

**Figure 6:**
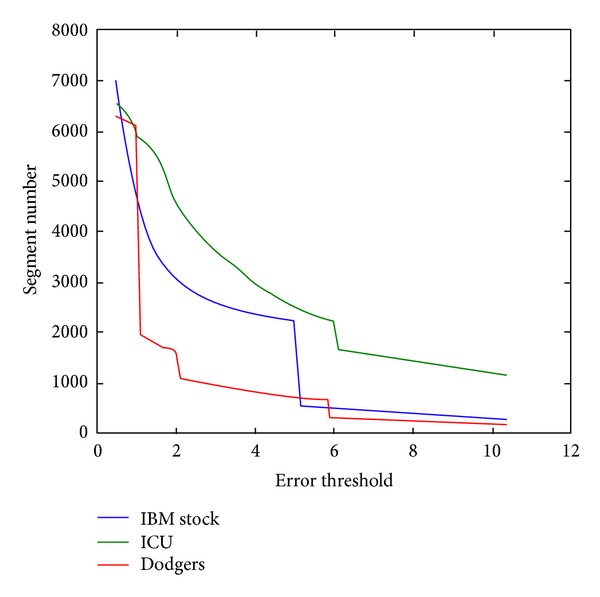
Relative of error threshold and segments number for BU.

*Problem of Getting Proper Number of Segments.* In Table 2, for the real datasets ChinaFLUX, BU fails to find a proper number of segments because this kind of data changes tremendously. This is the flaw of BU. One significant advantage of PRESEE over BU is that users do not need to set any threshold parameter. The parameter of BU is hard to set. A little deviation would generate a quite different number of segments. In order to find out the relationship between segment size and the parameter error threshold that BU requires, we run each dataset 100 times to find a proper parameter that can generate the same number of segments as PRESEE.  Figure 6 shows their relationship for BU. In the experiment, all three real datasets encounter a big drop of segments number when the error threshold increases. In particular, for Dodgers datasets, the number of segments drops from 6082 to 1958 when the value changes from 1.0 to 1.1, which is very significant. Further experiments show that, even we only increase the threshold with 0.001, the change is also tremendous. Such phenomenon indicates that we should be careful to the error threshold parameter of BU. Such puzzle can be well avoided by the user of PRESEE.

*Efficiency and Scalability.* We only test the relative speed of algorithms since the absolute speed (running time) is varied according to machines. The speed of synthetic-dataset-generated algorithm is used as a benchmark. It reflects the maximum processing speed that a certain running machine can reach.

**Figure 7:**
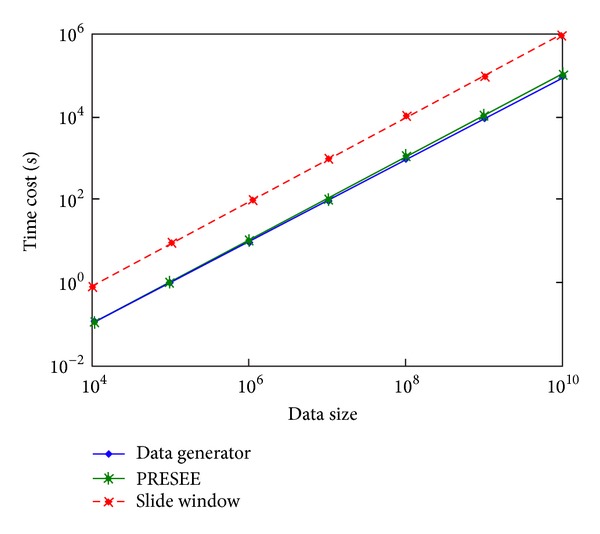
Efficiency and scalability of different algorithms.

**Figure 8:**
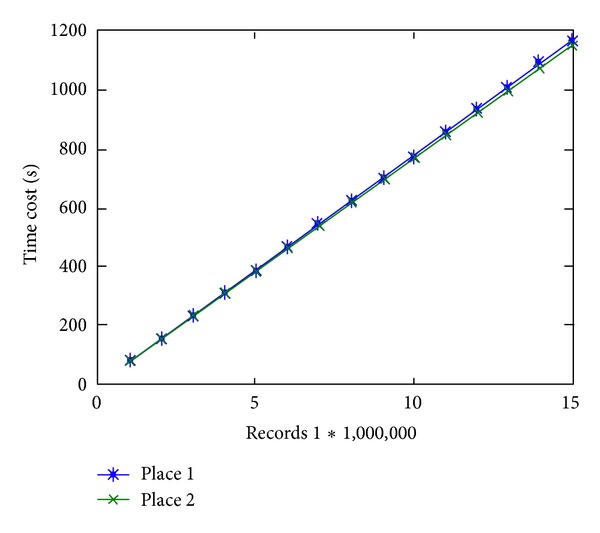
Efficiency of PRESEE for different datasets. Datasets are gathered from different places by ChinaFLUX.

Since the IBM stock, ICU, and Dodger datasets are too small and not suitable for horizontal comparison in efficiency and scalability test, we use real-time ChinaFLUX datasets and the synthetic datasets 1–4 and 9–11 in this experiment. At first, we compare the efficiency among data generator, SWBU, and PRESEE. The error rate threshold of SWBU set as 1.1 means that SWBU can generate comparatively the same number of segments with PRESEE.  Figure 7 shows the efficiency of different algorithms in logarithmic plot for synthetic datasets 1–4 and 9–11. It is certain that data generator owns the best speed since it just generates data without any extra processing. In this figure, we can find that the curve of PRESEE is very near to the curve of data generator and these two are far from the curve of SWBU (near one order of magnitude).  Figure 8 shows its efficiency on different datasets (datasets ChinaFLUX).  Figure 8 indicates that the efficiency would not be affected by characters of datasets.  Table 3 shows the relative speed of two segmenting algorithms' relative speed (with *t*_seg_ representing the time cost of segmenting algorithm and *t*_gen_ representing the time cost of data generator). PRESEE is just a little slower than data generator with the value 1.1311, while SWBU is nearly one order of magnitude slower. This is because PRESEE is an *O*(*n*) algorithm, but SWBU uses BU, an *O*(*n* log* n*) algorithm, as the base algorithm in slide window. With larger error rate threshold set, slower SWBU would run.

**Figure 9:**
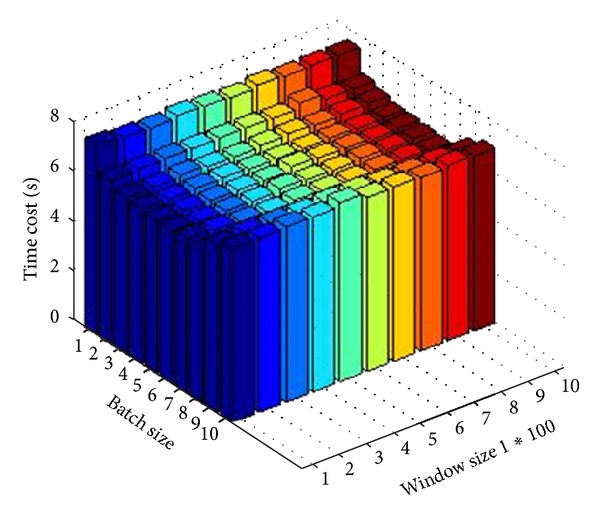
Relationship among efficiency, window size, and batch size.

*Features Affecting Algorithm Efficiency.* In this section, we do some experiments to explore the characteristic of our algorithm. There are two optional parameters for PRESEE: window size and batch size. Window size controls the number of points to process at once; batch size controls how many result segments are output per time. As is mentioned before, there is no necessary parameter in PRESEE, so the user can directly use default values for the two parameters. In order to see how the two parameters affect the algorithm's efficiency, we run synthetic dataset number 3 for 100 times, set window size from 100 to 1000 and batch size from 1 to 10.  Figure 9 shows the triple relationship among the efficiency, window size, and batch size. This figure indicates that the time cost decreases while the batch size increases, but if the batch size is too large, the efficiency of algorithm decreases. The reason is that more segments output per time can accelerate the speed of algorithm and let the slide window move faster at first. Gradually, the efficiency begins to decrease with segments size increases. There are two reasons why this phenomenon happens: firstly, the points in slide window do not have such many segments, the efficiency cannot increase forever. Secondly, the algorithm would cost extra time to identify the character points of each segment.

*Compress Rate.* There is no parameter to control the precision of result since MDL/MML owns the self-adaptive property. We do an experiment to see the *compress rate* (*compress rate* = *size of result*/*size of original dataset*) on different kinds of datasets. We choose ChinaFLUX datasets, IBM stock price, ICU and Dodgers datasets, and synthetic datasets 4–8 to do the experiments. The four real datasets are quite different in their appearance and they are different in cyclical/noncyclical, sharp/smooth, degree of noise, dimensions, and length. The synthetic datasets 4–8 have the same parameter on data size and range, and they are generated in the same way. The only difference is the degree of *sharpness*.

From Table 4, we can conclude that, for the same kind of data and with different *sharpness*, the compress rate is steady.

## 7. Conclusion and Future Work

In this paper, we have proposed a new algorithm for time-series stream segmenting that is parameter free, scalable and self-adaptive. We also undertake several sets of time-series experiments on a variety of time series data types and compare them with the state-of-the art algorithms to evaluate our algorithm. The empirical results prove that PRESEE can generate a proper number of segments for time-series streams. Moreover, it can handle large dataset up to gigabytes. Finally, the parameters of PRESEE would only affect the efficiency but not the segmenting result.

In the future, we plan to design a series of time-series stream mining algorithms under the pipeline framework. Those algorithms should be able to well concatenate to the segmenting algorithm. Another direction is to design the algorithms that can do time-series mining tasks across multiple streams in real time.

## Figures and Tables

**Algorithm 1:**
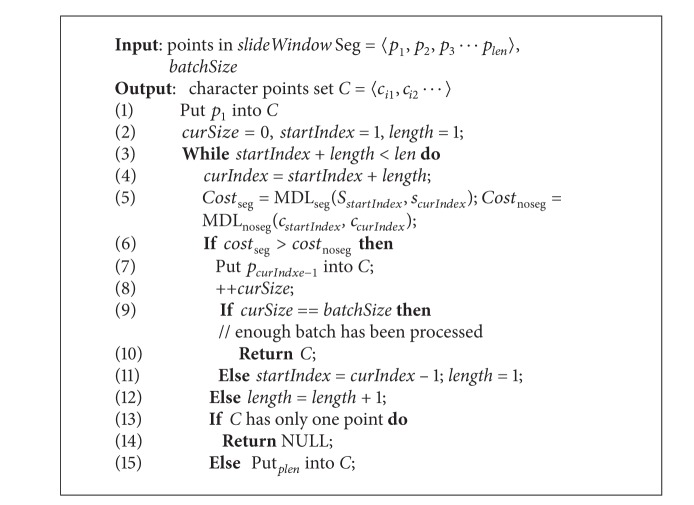
Segment in slide window.

**Algorithm 2:**
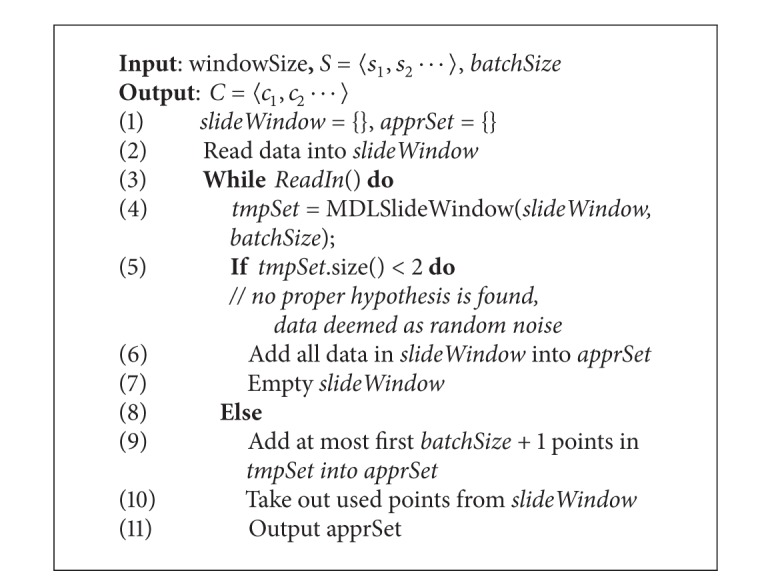
PRESEE.

**Table 1:** Parameters for synthetic datasets.

Number	Data size	Lower bound (lb)	Upper bound (ub)	Sharpness
1	10,000 (10 k)	0	3000	0.001
2	100,000 (100 k)	0	3000	0.001
3	1,000,000 (1 M)	0	3000	0.001
4	10,000,000 (10 M)	0	3000	0.001
5	10,000,000 (10 M)	0	3000	0.002
6	10,000,000 (10 M)	0	3000	0.0005
7	10,000,000 (10 M)	0	3000	0.0002
8	10,000,000 (10 M)	0	3000	0.0001
9	100,000,000 (100 M)	0	3000	0.00005
10	1,000,000,000 (1 G)	0	5000	0.00001
11	10,000,000,000 (10 G)	0	5000	0.00001

**Table 2:** Precision of datasets.

Number	Data size	Error rate (BU)	Error rate (PRESEE)
1	10,000 (10 k)	2.00201	2.58561
2	100,000 (100 k)	1.75349	2.45916
3	1,000,000 (1 M)	1.67456	4.03172
4	10,000,000 (10 M)	1.65483	2.28419
5	10,000,000 (10 M)	1.66074	2.28695
6	10,000,000 (10 M)	1.65577	2.28768
7	10,000,000 (10 M)	1.65978	2.28862
8	10,000,000 (10 M)	1.66243	2.28124
9	100,000,000 (100 M)	1.67212	2.29100
10	100,000,000 (1 G)	N/A	2.27872
11	100,000,000 (10 G)	N/A	2.27984
12	IBM stock price from 1.2.1961 to 1.6.2010 (12087 records)	1.57696	2.38069
13	ICU (7931 records)	0.696307	0.893313
14	Dodger (10082 records)	1.34914	1.08395
15	ChinaFLUX (38850980 records)	N/A	1.76781

**Table 3:** Relative speed of SWBU and PRESEE compared with data generated by the data generator.

Algorithm	Relative speed (*t*_seg_/*t*_gen_)
SWBU	9.7114
PRESEE	1.1311

**Table 4:** Compress rate of datasets.

Number	Data size	Compress rate
4	10,000,000 (10 M)	1 : 0.27548
5	10,000,000 (10 M)	1 : 0.27536
6	10,000,000 (10 M)	1 : 0.27537
7	10,000,000 (10 M)	1 : 0.27531
8	10,000,000 (10 M)	1 : 0.27536
12	IBM stock price	1 : 0.43779
13	ICU	1 : 0.48468
14	Dodgers	1 : 0.35540
15	ChinaFLUX	1 : 0.48194
